# PMMCT: A Parallel Multimodal CNN-Transformer Model to Detect Slow Eye Movement for Recognizing Driver Sleepiness

**DOI:** 10.3390/s25185671

**Published:** 2025-09-11

**Authors:** Yingying Jiao, Jiajia Zhang, Zhuqing Jiao

**Affiliations:** School of Computer Science and Artificial Intelligence, Aliyun School of Big Data, Changzhou University, Changzhou 213159, China; s23150812061@smail.cczu.edu.cn (J.Z.); jzq@cczu.edu.cn (Z.J.)

**Keywords:** driver sleepiness, slow eye movement, CNN-transformer, electrooculogram (EOG), electroencephalograph (EEG), multimodal fusion, sleep onset period (SOP)

## Abstract

Sleepiness at the wheel is an important contributor to road traffic accidents. Slow eye movement (SEM) serves as a reliable physiological indicator for the sleep onset period (SOP). To detect SEM for recognizing drivers’ SOP, a Parallel Multimodal CNN-Transformer (PMMCT) model is proposed. The model employs two parallel feature extraction modules to process bimodal signals, each comprising convolutional layers and Transformer encoder layers. The extracted features are fused and then classified using fully connected layers. The model is evaluated on two bimodal signal combinations HEOG + O2 and HEOG + HSUM, where HSUM is the sum of two single-channel horizontal electrooculogram (HEOG) signals and captures electroencephalograph (EEG) features similar to those in the conventional O2 channel. Experimental results indicate that using the PMMCT model, the HEOG + HSUM combination performs comparably to the HEOG + O2 combination and outperforms unimodal HEOG by 2.73% in F1-score, with average classification accuracy and F1-score of 99.89% and 99.35%, outperforming CNN, CNN-LSTM, and CNN-LSTM-Attention models. The model exhibits minimal false positives and false negatives, with average values of 5.2 and 0.8. By combining CNNs’ local feature extraction with Transformers’ global temporal modeling, and using only two HEOG electrodes, the system offers superior performance while enhancing wearable device comfort for real-world applications.

## 1. Introduction

**Figure 1:**
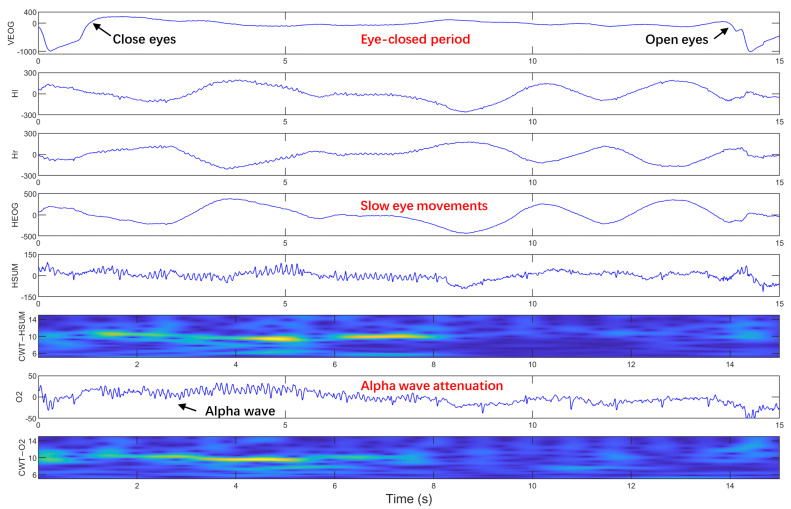
SEMs with lower frequency in longer eye-closed periods. Hl and Hr: two signal-channel HEOG electrodes at the outer canthi of both eyes. HEOG = Hl − Hr, HSUM = Hl + Hr. O2: occipital electrode. CWT-HSUM and CWT-O2 show the time-frequency energy distribution after continuous wavelet transform (CWT) with complex Morlet wavelet. The VEOG signal is the vertical EOG signal, which is the difference of signals from two signal-channel located above and below one eye.

Sleepiness at the wheel is a critical factor contributing to traffic accidents [1,2], making early detection of sleep onset essential for accident prevention. Sleep-related studies report that slow eye movement (SEM) is a reliable physiological indicator of the sleep onset period (SOP) [3,4], which refers to the transition from wakefulness to sleep [5,6,7]. SEMs emerge before sleep stage I, go through it, and decline progressively during sleep stage II [8]. Compared to rapid eye movements such as blinks and saccades during the alert state, SEMs are characterized by slow, rolling, sinusoidal, conjugate horizontal eye movements with relatively large amplitudes, recorded in horizontal electrooculogram (HEOG) signals, as shown in Figure 1. Pizza et al. demonstrated that during wakefulness after sleep onset (WASO), SEMs occupy approximately 61 ± 13% of the total duration [9].

**Figure 2:**
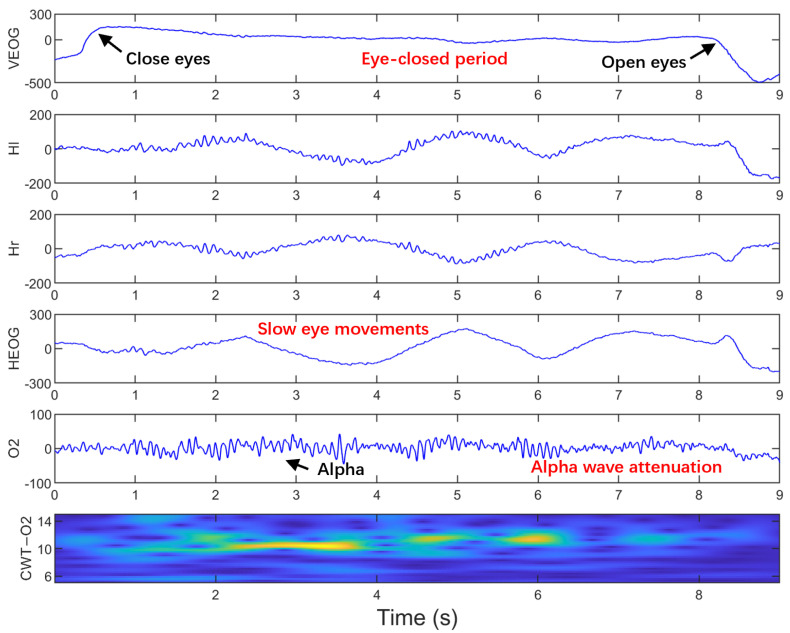
SEMs with higher frequency in shorter eye-closed periods. Hl and Hr: two signal-channel HEOG electrodes at the outer canthi of both eyes. HEOG = Hl − Hr. O2: occipital electrode. CWT-O2 shows the time-frequency energy distribution after continuous wavelet transform (CWT) with complex Morlet wavelet. The VEOG signal is the vertical EOG signal, which is the difference of signals from two signal-channel located above and below one eye.

However, SEMs have received limited attention in driver-fatigue detection research. Unlike sleep processes, the driving process involves primarily eyes-open phases, with only brief periods of eye-closure. Sleep research has demonstrated that SEMs show a straight relationship with electroencephalogram (EEG) changes during the SOP. Specifically, EEG power exhibits a negative correlation with SEMs in the 1–14 Hz range, while showing a positive correlation in the 15–30 Hz range [5,10]. Our previous work found that the SEMs often occur during the eye-closed period, which is covered by the SEMs at a higher rate and shows higher duration distribution than those without SEM, indicating higher sleepiness levels for the occurrence of the SEMs [10]. In our simulated driving, the occurrence of SEMs is often accompanied by the attenuation of the EEG alpha wave, as demonstrated in Figure 1 and Figure 2. Attenuation of the EEG alpha wave is considered the most reliable physiological marker of SOP in sleep research [6,7]. However, EOG signals provide higher amplitude and signal-to-noise ratios compared to EEG signals, making them more suitable for practical applications [11]. Therefore, it is feasible and practical to identify the driver’s SOP by detecting SEM. Furthermore, combining EEG with HEOG signals is expected to enhance SEM detection accuracy.

Early SEM detection methods mainly rely on the characteristics of SEM in HEOG signals, such as frequency, amplitude, and rate. These methods employ statistical analysis and signal processing techniques to extract distinguishing features and construct discriminant functions based on these features to achieve detection tasks [12,13,14]. Recent advancements by Jiao et al. have introduced machine learning frameworks to transform SEM detection into a binary classification problem [10,15]. Their initial work extracted handcrafted features from HEOG signals and used a support vector machine (SVM) for classification [15]. They later extracted complementary EEG features in addition to HEOG signal features, developing a bimodal long short-term memory (LSTM) to process these features for improved SEM detection [10]. However, the aforementioned methods rely on handcrafted feature extraction and domain expertise, which limits the ability to perform and generalize [16].

Deep learning methods automatically learn feature representations through multiple network layers, enabling end-to-end processing from raw data to final predictions [17,18]. Convolutional Neural Networks (CNNs) have demonstrated powerful feature extraction capabilities through convolutional layers. Yildirim et al. proposed a 19-layer 1D-CNN model, achieving high accuracies for two to six sleep classes in sleep stage classification [19]. Although CNNs are highly effective at learning local, shift-invariant features through convolution, their fixed receptive fields and lack of inherent recurrence limit their ability to model long-range temporal dependencies and capture dynamic contextual variations in sequential signals [20,21]. In contrast, LSTM networks effectively capture sequential and temporal patterns using gated memory cells [22]. To leverage the strengths of both architectures, CNN-LSTM hybrid models have been developed. For instance, Lee et al. introduced an LSTM-CNN approach to detect drowsiness using EEG signals, achieving 85.6% accuracy for three-class classification and a 0.94 F1-score for binary classification [23]. Supratak et al. introduced DeepSleepNet, a CNN-bidirectional LSTM framework designed for automatic sleep stage classification from raw single-channel EEG, achieving state-of-the-art accuracy and F1-scores [24]. Similarly, Korkalainen et al. utilized a multi-scale CNN combined with LSTM networks to extract both local and temporal features from single-channel frontal EEG data, improving sleep stage classification results in patients with suspected obstructive sleep apnea [25]. However, LSTM-based models may experience information loss when training on long sequences [26,27]. To overcome this limitation, the Transformer architecture was introduced, employing multi-head self-attention mechanisms to effectively capture long-range dependencies in sequential data [28]. Li et al. developed a Time–Frequency Transformer (TFormer) for EEG-based fatigue recognition, leveraging multi-head cross-attention to fuse time-frequency features and achieving state-of-the-art results [29]. Eldele et al. proposed AttnSleep, a novel sleep stage classification model that mainly integrates a multi-resolution CNN for feature extraction and a temporal context encoder based on multi-head self-attention, achieving state-of-the-art results on single-channel EEG signals [21]. Dai et al. employed Transformer encoders for feature extraction and fusion from multichannel polysomnography (PSG) data, attaining high accuracy in sleep stage classification [30]. Qu et al. developed a fast, end-to-end deep learning framework combining CNNs with a transformer’s self-attention module to capture global temporal correlations, obtaining state-of-the-art outcomes for single-channel EEG-based sleep staging [31]. Ding et al. proposed a CNN-Attention model for single-channel EEG-based driver drowsiness detection, achieving 97.09% accuracy with real-time on a wearable headband system [32].

Parallel to these advances in physiological signal analysis, Transformer-based Large Language Models (LLMs) and Multi-modal LLMs (MLLMs) have demonstrated strong capabilities in integrating heterogeneous inputs and enabling context-aware reasoning. In vision-based driver monitoring, recent studies have shown that a reasoning-chain LLM can provide explainable distracted driving classification [33], an LLM-driven agent can deliver personalized multimodal safety warnings [34], and prompt-guided MLLMs can achieve accurate driver state recognition without fine-tuning [35]. Although these approaches focus on visual signals, they highlight a broader paradigm shift toward more intelligent and interpretable Transformer-based systems. This trend directly motivates the integration of a Transformer module into our PMMCT framework to enhance robust physiological signal analysis.

Accurate SEM detection is crucial for driving safety, but previous methods often suffer from high false detection rates and require multiple channels, limiting real-world deployment. To overcome these challenges, we propose a Parallel Multimodal CNN-Transformer (PMMCT) model that processes bimodal signals in parallel through two feature extraction modules. To validate the effectiveness of the proposed model, it is evaluated on two bimodal signal combinations HEOG + O2 and HEOG + HSUM, where HSUM is the sum of two single-channel HEOG signals. The results are compared with the unimodal HEOG method and other baseline models. The contributions are summarized as follows:

(1) We propose a PMMCT model that combines the advantages of convolutional modules and the self-attention mechanism in Transformers, enabling the effective capture of both local and global features of the signals.
(2) We develop an efficient parallel feature extraction and fusion strategy for bimodal signals, which improves detection performance with minimal false positives and false negatives.
(3) We validate that the HEOG+HSUM combination applied to the PMMCT model yields comparable performance to the HEOG + O2 combination, enabling accurate SEM detection with only dual single-channel HEOG electrodes, which enhances practicality in real-world driving scenarios.
(4) We developed SEMData, a publicly available dataset comprising dual single-channel HEOG and single-channel EEG (O2) data from 10 participants during simulated driving to support SEM detection research.

## 2. Materials

### 2.1. Experimental Settings

To ensure the authenticity and practicality of experimental data, all physiological and behavioral signals were synchronously collected in a self-developed simulated driving device. The experiment involved 10 subjects (6 females, 4 males; age 24 ± 3 years) with long-term napping habits (>1 year). Participants engaged in simulated driving within a real engine-free vehicle, facing an LCD screen displaying a monotonous straight four-lane highway scene synchronized with steering wheel and throttle inputs. Daytime sleepiness was assessed via the Epworth Sleepiness Scale (ESS) ranging from 0 to 24 points [36], with an average score of 11 ± 1.5, indicating mild-to-moderate levels. The experiment commenced 30 min before their habitual naptime, lasting 1.5 h to induce mild sleep deprivation, while minimizing body movements to reduce physiological signal artifacts.

### 2.2. Data Acquisition

**Figure 3:**
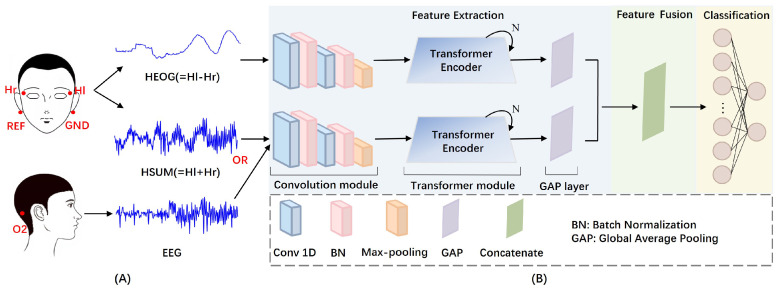
Overall framework of PMMCT model for SEM detection: (**A**) electrode placement. Hr and Hl: two signal-channel HEOG electrodes at the outer canthi of both eyes. REF: refer ence electrode. GND: ground electrode. O2: occipital electrode. (**B**) model architecture with sequential feature extraction, feature fusion, and classification.

The ESI NeuroScan system was used for data acquisition, with all sensors arranged according to standard protocols to maximize signal-to-noise ratio. As shown in Figure 3A, HEOG electrodes were placed at the outer canthi of both eyes (Hl and Hr) to accurately capture horizontal eye movements, while an O2 electrode was positioned over the occipital region to record EEG activity. The innovative HSUM signal, defined as the sum of Hl and Hr, was introduced to evaluate the feasibility of extracting EEG features with simplified hardware configurations. All signals were recorded at a 500 Hz sampling rate, with reference and ground electrodes placed behind the mastoids. The reference and ground electrodes were positioned behind the mastoids on both sides of the ears. Additionally, a high-resolution camera was installed to monitor and record facial movements continuously. The SCAN software (Version: SCAN 4.3) was used to synchronize the physiological signals and video data, enabling precise correspondence verification between eye movements and signal events.

### 2.3. Data Preprocessing

In the NeuroScan system, each electrode (Hr, Hl, and O2) was preconfigured with a 0.1–30 Hz band-pass filter to restrict the acquired signal bandwidth within this range. Considering that the frequency range of SEMs in HEOG signals is 0.2–0.5 Hz, we applied median filtering (with window length parameter set to 10) to the HEOG signal for smoothing. For the O2 or HSUM signals, a 1D Discrete Wavelet Transform (DWT) with the Daubechies 2 mother wavelet is applied, with the level of the decomposition set to 10 [37]. The reconstructed approximation signal at the 10th level is then subtracted from the original signals to effectively remove baseline drift. This process preserves key signal features while removing noise and drift, improving overall quality.

### 2.4. Visual Labeling of SEMs

**Figure 4:**
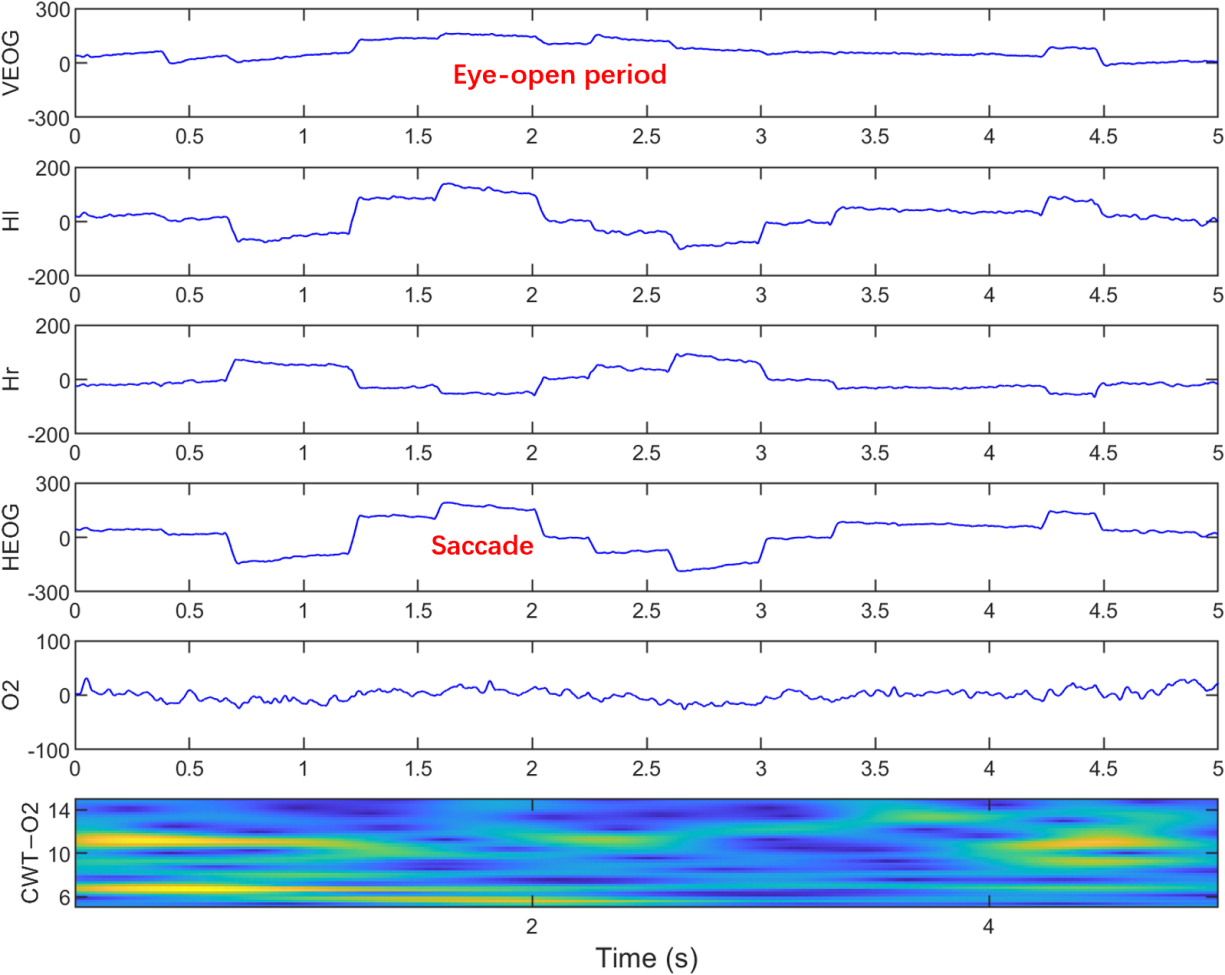
Saccades during the alert state with eyes open.

In sleep research, SEMs were visually marked based on criteria established in previous literature [38,39]. Specifically, SEMs must simultaneously meet the following characteristics: (1) synchronous and slow sinusoidal excursions with opposite phases in two single-HEOG channels (Hl and Hr); (2) signal frequency between 0.2–0.6 Hz lasting over 2 s; (3) amplitude range of 20–200 μV; (4) onset time difference between channels no greater than 300 ms; (5) no obvious artifacts in signals. These same criteria are used to label SEMs that occur during simulated driving. The SEM waveform is clearly different from the saccade that occurs during the alert state (eyes open), as shown in Figure 4.

In our simulated driving experiments, SEMs predominantly occur during the eye-closed period, which is the period between the upward trend line caused by closing eyes and the downward trend line caused by re-opening eyes, as shown in Figure 1 and Figure 2. SEMs have a relatively high frequency in the early stages of simulated driving as shown in Figure 2, and a relatively low frequency in the later stages when more sleepy (longer eye-closed periods) as shown in Figure 1. The occurrence of SEMs is often accompanied by the alpha wave’s attenuation, which is observed to appear synchronously on two single-channel HEOG signals and O2 signals. Additionally, the HSUM signals (=Hl + Hr) exhibited EEG spectral features similar to those in the O2 channel during eye closure. This similarity is supported by their alpha-band (6–14 Hz) time-frequency energy distributions, analyzed via continuous wavelet transform (CWT) with complex Morlet wavelets (Figure 1, CWT-HSUM and CWT-O2).

## 3. Methods

The problem of detecting SEMs is transformed into a binary classification problem. To address this, we propose a parallel multimodal CNN-Transformer (PMMCT) model to process synchronized bimodal signals. Figure 3B illustrates the overall framework of the PMMCT model, containing three sequential stages: (1) feature extraction, (2) feature fusion, and (3) classification. In the feature extraction stage, bimodal signals are respectively fed into two parallel blocks, each composed of a convolutional module, a transformer module, and a global average pooling (GAP) layer. In the feature fusion stage, the two extracted feature sequences from the parallel blocks are concatenated to form a fused feature representation. Finally, this feature representation is passed through the fully connected layers for classification. The model is evaluated in a subject-specific manner, with a separate model trained for each participant.

### 3.1. Feature Extraction

In the feature extraction stage, two parallel blocks process bimodal signals synchronously. In each block, the convolution module first extracts local features. Then, the features and their positional encoding are added and fed into the transformer module. Finally, a GAP layer is applied to the transformer output.

#### 3.1.1. Convolution Module

The convolution module consists of two convolutional layers and a max pooling (MP) layer. Each convolutional layer contains a convolution operation, a batch normalization (BN) operation, and a Rectified Linear Unit (ReLU) activation function. When performing a 1D convolution, a convolution kernel with a length of *L* and a width of *C* moves in one direction from the beginning to the end of the time series. The width *C* of the kernel is the same as the width of the time series, while the length *L* can vary. At each moving step, the elements of the convolution kernel are multiplied by the corresponding elements in the local region covered by the kernel in the time series, which can be expressed as follows:

(1) $$y_{i}^{\left(m+1\right)}\left(j\right)=W_{i}^{m}\cdot X^{m}\left(j\right)+b_{i}^{m}$$

where *j* is the index of the *j*-th moving step, *i* is the index of the kernel, $W_{i}^{m}$ and $b_{i}^{m}$ are the weight and bias of the *i*-th kernel in layer *m*, $X^{m}\left(j\right)$ denotes the *j*-th local region in layer *m* with the same size as the kernel. The BN operation is applied after convolution to reduce internal covariate shifts. Performing the BN operation before the activation function normalizes the input distribution, enabling effective non-linear transformations and mitigating the vanishing gradient problem [40]. After two convolutional layers, the MP layer is used for downsampling, which preserves key features while reducing computational complexity.

#### 3.1.2. Transformer Module

**Figure 5:**
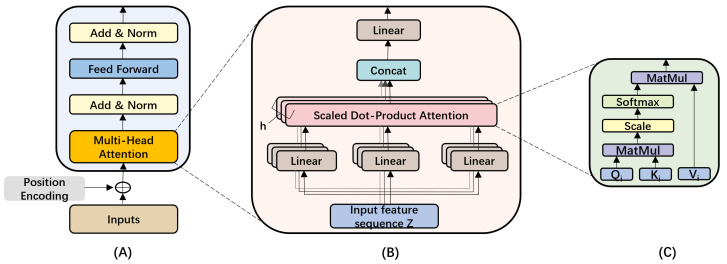
Architecture of (**A**) transformer encoder, (**B**) multi-head attention, and (**C**) scaled dot-product attention.

The Transformer module consists of two identical encoder layers (in Figure 3, N=2), each has two main components: a multi-head attention (MHA) layer and a feed-forward neural network (FFN), as shown in Figure 5A. In addition, a residual and normalization layer is added respectively after the MHA layer and the FFN to improve model stability and robustness. As the encoder discards recurrence [30], positional encodings for the features from the convolution module are added to the input of the encoder layer to introduce temporal information.

**Multi-Head Attention (MHA)** The attention mechanism used in MHA is scaled dot-product attention, as shown in Figure 5B,C, which generates the output sequence by capturing relationships between elements at different positions in the input feature sequence [41,42]. MHA consists of *h* (*h* = 2) parallel scaled dot-product attention modules, each performing attention computation independently. Specifically, in the *i*-th scaled dot-product attention module, the input feature sequence *Z* is mapped into Query ($Q_{i}$), Key ($K_{i}$), and Value ($V_{i}$) vectors via linear transformations, which is expressed in Equation (2):

(2) $$Q_{i}=Z\cdot W_{i}^{Q},\;K_{i}=Z\cdot W_{i}^{K},\;V_{i}=Z\cdot W_{i}^{V}$$

where $Z\in R^{l\times d}$ is the input with length *l* and dimension *d*, $Q_{i}\in R^{l\times d_{k}}$, $K_{i}\in R^{l\times d_{k}}$, $V_{i}\in R^{l\times d_{v}}$ represent the query, key, and value matrices. $W_{i}^{Q}\in R^{d\times d_{k}}$, $W_{i}^{K}\in R^{d\times d_{k}}$, $W_{i}^{V}\in R^{d\times d_{v}}$ are the learnable weight matrices. Here, i∈[1,h], where *h* is the number of attention heads. Then, the *i*-th attention head $H_{i}$ is computed based on the above $Q_{i}$, $K_{i}$, and $V_{i}$, as expressed in Equation (3):

(3) $$H_{i}=\mathrm{Attention}\left(Q_{i},K_{i},V_{i}\right)=\mathrm{Softmax}\left(\frac{Q_{i}K_{i}^{T}}{\sqrt{d_{k}}}\right)V_{i}$$

The final MHA output is obtained by concatenating the results of all heads and applying a linear transformation:

(4) $$\tilde{Z}=\mathrm{Concat}\left(H_{1},H_{2},\ldots ,H_{h}\right)\cdot W^{O}$$

where $W^{O}\in R^{hd_{v}\times d}$ is the learnable weight matrix.

**Feed-Forward Neural Network (FFN)** The output of the MHA layer is fed into FFN, which consists of two fully connected layers. The first layer applies the ReLU activation function to introduce non-linearity into the model. The FFN is designed to perform nonlinear transformations of the embedding vectors. The specific formula is:

(5) $$\mathrm{FFN}\left(x\right)=\max\left(0,xW_{1}+b_{1}\right)W_{2}+b_{2}$$

where $W_{1}\in R^{d\times d_{\mathrm{ff}}}$ and $b_{1}\in R^{d_{\mathrm{ff}}}$ are the weight matrix and bias vector of the first linear transformation, $W_{2}\in R^{d_{\mathrm{ff}}\times d}$ and $b_{2}\in R^{d}$ are the weight matrix and bias vector of the second linear transformation and $d_{\mathrm{ff}}$ is the internal dimension.

**Residual and Normalization Layer** The encoder layer has two *Add* & *Norm* layers, which add the output of the previous layer to the input of that layer through a residual connection, and then normalize the sum. This operation can be expressed as:

(6) $${\tilde{Z}}_{\mathrm{mid}}=\mathrm{LayerNorm}(Z+\tilde{Z})$$

(7) $$O=\mathrm{LayerNorm}({\tilde{Z}}_{\mathrm{mid}}+\mathrm{FFN}({\tilde{Z}}_{\mathrm{mid}}))$$

where LayerNorm refers to applying layer normalization.

#### 3.1.3. Global Average Pooling (GAP)

The GAP layer compresses each feature channel by averaging all temporal embeddings, reducing the channel-wise output to a single scalar value [43]. This pooling method not only reduces model parameters but also adaptively integrates important information across the sequence, enhancing the model’s robustness.

### 3.2. Feature Fusion

In this stage, the extracted features of the two signals are fused through the concatenate operation to form a fused feature representation, achieving effective integration of multimodal information.

### 3.3. Classification

We adopt two fully connected layers in this stage. The feature representation passes through the first fully connected layer with a ReLU activation function, followed by a dropout layer. The output is then produced using Softmax for classification with weighted cross-entropy loss as follows:

(8) $$L=-\sum_{i=1}^{2}w_{i}y_{i}\log({\hat{y}}_{i})\;$$

where $y_{i}$ is the corresponding target label (in one-hot encoding), ${\hat{y}}_{i}$ is the *i*-th output prediction, and $w_{i}$ represents the weight assigned to class *i*. To address the class data imbalance in the dataset, we implement a weighting strategy [44,45], where the class weight is calculated as

(9) $$w_{i}=\frac{N}{c\times N_{i}}$$

where *N* is the total number of samples, $N_{i}$ is the number of samples in class *i*, and *c* is the number of classes. This weighted approach balances model training by assigning higher weights to minority classes and lower weights to majority classes, thereby improving the model’s ability to learn from imbalanced data.

## 4. Experimental Results

In this section, we first introduce the experimental setup and compare the PMMCT model with several baseline models. We then evaluate the effectiveness of multimodal configurations. Additionally, we perform a sensitivity analysis of the transformer parameters to assess the specific impact of different parameter settings on model performance.

### 4.1. Data Preparation

Following the visual labeling criteria (Section 2.4), we first marked SEM and non-SEM epochs in each subject’s data. A 3-s sliding window with a 0.1 s step size was then applied to extract standardized samples from both SEM and non-SEM epochs, creating two-class window data samples for each subject. The labeled epoch, referred to as SEM_epoch_mark, is publicly available at https://github.com/DriverSleepinessJiao/SEM_PMMCT/tree/main/SEMdata/SEM_epoch_mark (accessed on 8 September 2025) to support reproducible research. Due to the sampling rate of 500 Hz, each window data sample should be 1 × 1500. To reduce the amount of data computation, each window data sample was further downsampled to 1 × 500. For each subject, the former 70% of the data was used for training and validation, with five-fold cross-validation applied for hyperparameter optimization, while the latter 30% was reserved for independent testing [41]. To ensure data quality, noise-corrupted segments, and ambiguous waveforms were excluded.

### 4.2. Experimental Setup

#### 4.2.1. Running Environment

The PMMCT model was implemented using the Keras 2.6.0 deep learning framework in Python 3.9, with training executed on a GeForce RTX 3060 GPU (NVIDIA Corporation in Santa Clara, CA, USA) with 12.0 GB of dedicated memory. The model was trained using the Adam optimizer with hyperparameters $\beta_{1}=0.9$ and $\beta_{2}=0.999$ to minimize the categorical cross-entropy loss function [46]. The initial learning rate was set to 5 × 10^−4^ and was reduced adaptively by a factor of 0.2 if the validation loss did not improve for 3 consecutive epochs, down to a minimum of 1 × 10^−6^. The model was trained with a minibatch size of 128, and an early stopping mechanism was implemented to halt training if the validation accuracy did not improve after 10 epochs.

#### 4.2.2. Compared Algorithms

To comprehensively evaluate the proposed model, we compare it with three baseline models: CNN, CNN-LSTM, and CNN-LSTM-Attention. All models employ a parallel structure for processing bimodal signals and use weighted cross-entropy loss to address class imbalance with weights adjusted by class distribution. The CNN model is based on a basic parallel CNN structure; the CNN-LSTM model extends the CNN model with LSTM layers for temporal modeling; the CNN-LSTM-Attention model further integrates an attention mechanism into CNN-LSTM to enhance the capture of key features. Additionally, we compare our model with the Bimodal-LSTM proposed by Jiao et al. [10], which manually extracts features from bimodal signals.

#### 4.2.3. Hyperparameters Tuning

To determine the optimal hyperparameters for the convolution module while keeping all other parameters fixed, we employ a grid search combined with five-fold cross-validation [47]. The adjusted hyperparameters include the kernel sizes for the two convolutional layers applied to both bimodal signals. Each signal is processed by two convolutional layers, and for each layer, the kernel sizes are selected from [50, 150, 250], and for the models containing LSTM layers, the number of LSTM units is selected from [50, 100, 150]

#### 4.2.4. Evaluation Metrics

We adopted True positive (TP), True negative (TN), False positive (FP), False negative (FN), Precision (= TP/(TP + FP)), Recall (= TP/(TP + FN)), Accuracy (= (TP + TN)/(TP + TN + FP + FN)), and F1-score (= 2 × (Precision × Recall)/(Precisionm+Recall)) as evaluation metrics.

### 4.3. Cross-Correlation Analysis

**Figure 6:**
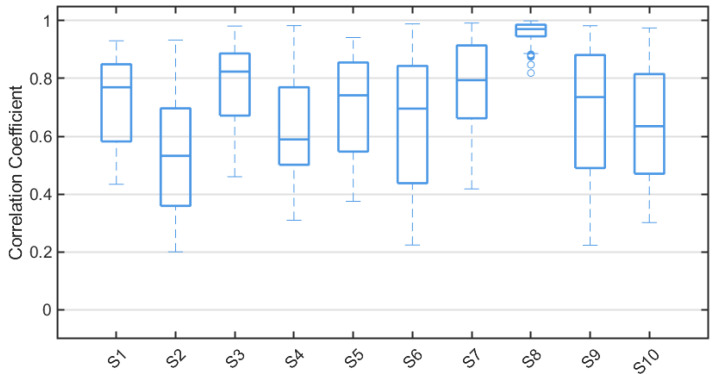
Distribution of Pearson correlation coefficients between the alpha-band wavelet energy curves of the O2 and HSUM signals for all samples in each subject.

For each subject, the labeled SEM and non-SEM epochs were segmented into non-overlapping 3-s window samples. For each pair of O2 and HSUM signals in a sample, the continuous wavelet transform (CWT) based on the complex Morlet wavelet (scale = 1024) was applied to extract the wavelet coefficient matrix $M\in C^{F\times T}$ within the alpha-band frequency range, where *T* denotes the time dimension. Along the time dimension, the mean of the wavelet coefficient matrix *M* across the frequency dimension is computed, resulting in a time-varying wavelet energy curve. This procedure generated paired wavelet energy curves for the O2 and HSUM signals in each sample, and the Pearson correlation coefficient between the two curves was computed. Figure 6 illustrates the distribution of correlation coefficient values between the wavelet energy curves of the O2 signal and the HSUM signal for all samples from each subject, where values exceeding twice the standard deviation of all sample correlation coefficients have been removed. It can be observed that the median correlation coefficient exceeds 0.7 for most subjects, indicating a high correspondence between the HSUM and O2 signals in the alpha band.

### 4.4. Scoring Performance

Table 1 presents the performance metrics of the PMMCT model across the 10 subjects under the conditions where the optimal parameters are obtained by subject-specific hyperparameter tuning. The model maintains consistently high accuracy across all subjects, with an average accuracy of 99.89 ± 0.13%. The F1-scores demonstrate strong reliability, with an average F1-score of 99.35 ± 0.62%. The model also demonstrates high precision, with S09 achieving a perfect 100% precision. The recall is similarly outstanding, with an average recall of 99.70 ± 0.40%, and five subjects (S01, S02, S03, S07, and S08) achieving perfect recall scores of 100%. Subject S04 shows slightly lower precision than the others but maintains high accuracy (99.53%) and recall (99.86%).

To evaluate the practical value of the proposed model, we analyze the FP and FN results based on the distribution of positive and negative samples in the test set for each subject as shown in Table 1. The proposed model demonstrates minimal FP across most subjects, typically fewer than 5, indicating that it rarely misclassifies non-SEM samples as SEM. Regarding FN, which represents instances where SEM samples are misclassified as non-SEM, the model demonstrates consistently low errors, with a maximum value of 2. This indicates that the model can detect nearly all SEM samples, meeting the stringent requirements for practical applications.

**Figure 7:**
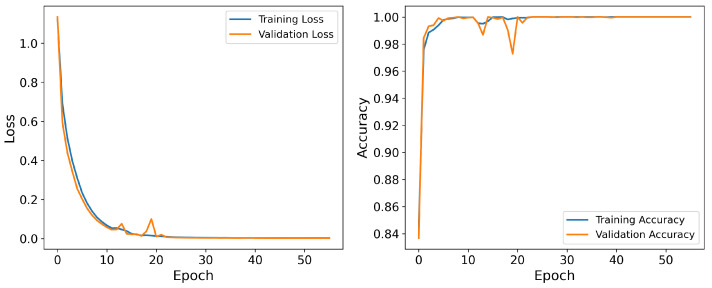
Accuracy and loss during training of PMMCT model for a randomly selected subject (S04).

Figure 7 illustrates the loss and accuracy curves during the training process, showing that the model converges quickly after a few epochs and reaches a stable state. During training, the accuracy of the training set steadily increases while the loss decreases. After several epochs, the accuracy and loss on the validation set stabilize, indicating that the model demonstrates robustness in preventing overfitting.

**Figure 8:**
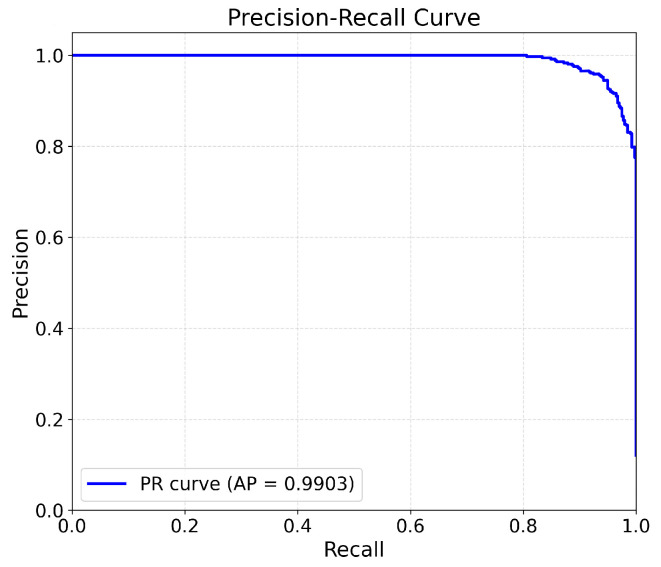
Precision–Recall (PR) curve for the imbalanced test data of a randomly selected subject (S04).

Moreover, we employ the Precision–Recall (PR) curve to evaluate the discriminative ability of the optimal PMMCT model on the testing data. As shown in Figure 8, the PR curve maintains high precision even at high recall levels and corresponds to an area under the curve (AUC) of 0.9903 for S04. These results demonstrate the robust classification capability of the proposed model.

### 4.5. Compared Results

Table 2 presents the average accuracy, F1-score, and their standard deviations across different parameter combinations. In terms of accuracy, the PMMCT model attains above 98.5% across all subjects, with S07 recording the highest value of 99.76 ± 0.28%. For F1-scores, the proposed model exceeds 95% in most subjects, with the peak score at 99.31 ± 0.75% for S08. In contrast, the CNN model’s accuracy fluctuates between 95% and 99%, with its F1-scores generally lower than those of the proposed model, the CNN-LSTM model shows large variations, with F1-scores under 85% in several subjects, although CNN-LSTM-Attention model yields higher scores through the attention mechanism, it still does not surpass the proposed model.

Under the optimal parameter configurations (Table 3), the test results of the PMMCT model record accuracies above 99.53%, with an average accuracy of 99.89 ± 0.13% and F1-score of 99.35 ± 0.62%, as shown in Table 4. In comparison, the baseline models CNN, CNN-LSTM, and CNN-LSTM-Attention show more instability in results: the average accuracies were 98.64 ± 1.77%, 97.24 ± 2.89%, and 99.25 ± 0.72% respectively, and the average F1-scores were 93.87 ± 5.44%, 85.52 ± 11.30%, and 96.00 ± 2.83% respectively. Additionally, as shown in Table 5, the PMMCT model with automatic feature extraction yields a 1.93% increase in classification accuracy and a 9.57% increase in F1-score compared to the Bimodal-LSTM model with manual feature extraction. The CNN model also surpasses Bimodal-LSTM, confirming the advantage of end-to-end feature learning. Notably, Bimodal-LSTM slightly outperforms CNN-LSTM, indicating that manual features encode temporal cues, whereas the LSTM module contributes little when trained end-to-end. In contrast, PMMCT shows a clear improvement by explicitly modeling global dependencies through the Transformer architecture. Specifically, the proposed model reaches the lowest FP and FN compared to all other models, demonstrating its superior feature extraction capability and highlighting its advantages in practical applications. The experimental results indicate that the PMMCT model outperforms the aforementioned baseline models on all metrics in SEM detection tasks. The average results in Table 2 show that the model attains high accuracy and F1-scores, with small standard deviations, indicating good stability across different subjects. The test results in Table 4 and Table 5 further validate the superiority of the proposed model.

### 4.6. Modality Analysis

**Figure 9:**
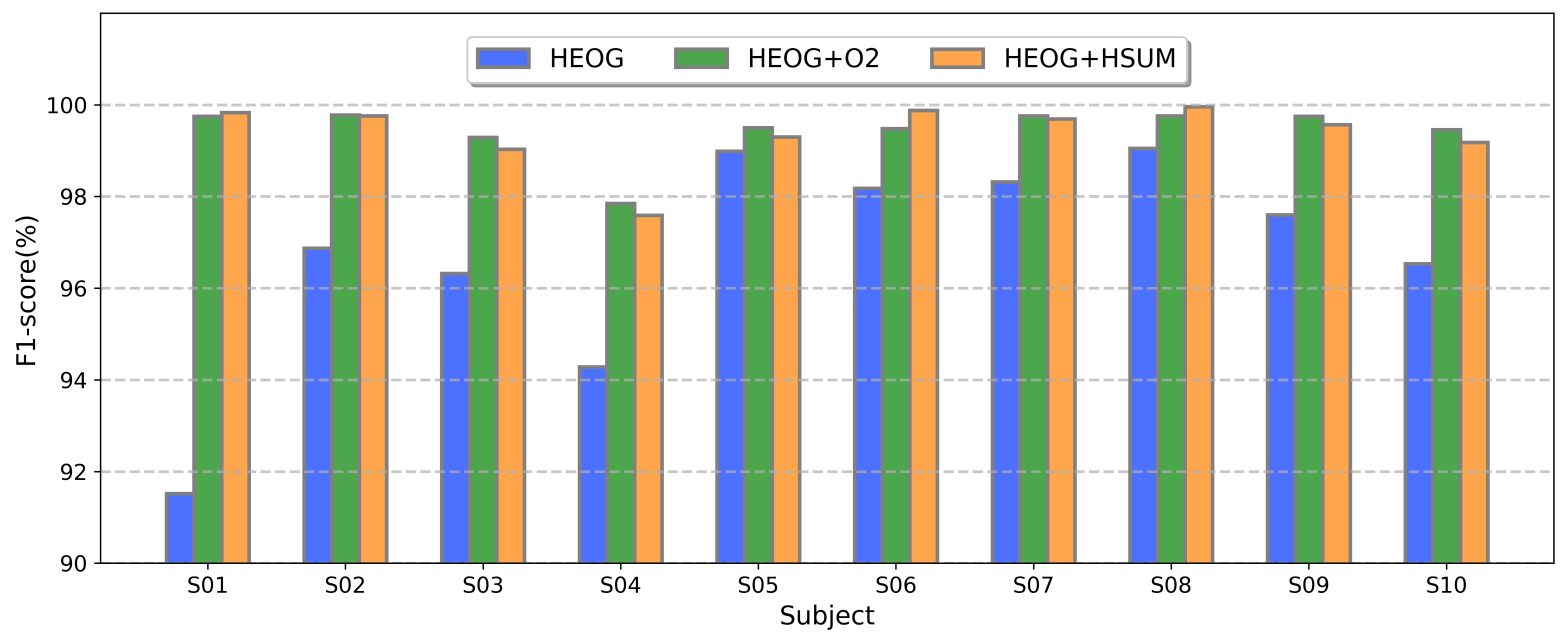
Performance comparison of different configurations for detecting SEMs: unimodal HEOG, HEOG + O2 combination, and HEOG+HSUM combination.

Figure 9 compares three kinds of modality configurations for SEM detection using the PMMCT model: the unimodal HEOG, the bimodal combination HEOG + HSUM, and the bimodal combination HEOG + O2. Results indicate that both bimodal combination configurations outperformed the unimodal HEOG across all subjects. The most significant improvement is observed in subject S01, where both bimodal configurations record F1-scores approximately 9% higher than the unimodal HEOG. On average, the HEOG + HSUM combination improves F1-scores by 2.75% compared to the unimodal HEOG. It is worth noting that the HEOG + HSUM and HEOG + O2 combinations demonstrate similar outcomes, with F1-scores surpassing 98% in most subjects. This similarity provides the foundation for our multimodal framework, where we utilize either EEG-related features from HSUM signals or O2-channel EEG features as complementary information to HEOG signals.

The comparable SEM detection performance between HEOG + HSUM and HEOG + O2 combinations supports that HSUM captures EEG features critical for SEM detection, consistent with their shared alpha-band characteristics (Figure 1). Notably, while the HEOG + HSUM combination only requires electrodes placed on both sides of the eyes, the HEOG + O2 combination needs an additional O2 electrode. The minimal performance gap across subjects between these two combinations suggests that HSUM can effectively serve as an alternative to O2-channel EEG features in multimodal SEM detection, reducing electrode complexity. These results also demonstrate the value of incorporating EEG features, whether from direct EEG signals in the O2 channel or derived HSUM signals, in enhancing SEM detection performance.

### 4.7. Parameter Sensitivity Analysis

Given that our model utilizes a backbone with Transformer encoders for feature extraction, we analyze the impact of the number of attention heads (*H*) and encoder layers (*N*) on model effectiveness using five-fold cross-validation. In each experiment, all parameters are kept constant except the one that is being tested. For a feature dimension of 64, we evaluate models with *H* = 1, 2, 4, 8, 16 and *N* = 1, 2, 4, 6, 8, where *H* must be divisible by the feature dimension to ensure equal division of attention.

**Figure 10:**
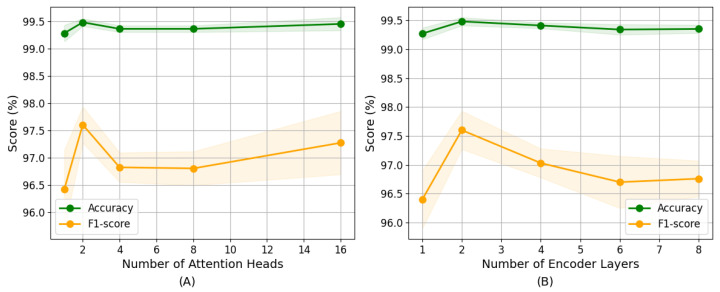
The impact of two parameters on model performance: (**A**) the number of attention heads, (**B**) the number of encoder layers (mean ± standard deviation over five-fold cross-validation for a randomly selected subject (i.e., S04)).

The results in Figure 10A indicate stable performance across different attention heads, with accuracy around 99.4% and F1-scores ranging between 96.5% and 97.5%. Increasing *H* from 1 to 2 markedly improved results, suggesting enhanced feature representation capabilities. However, further increasing *H* results in performance decline, particularly in the F1-score. The increase of *H* leads to the reduction of the feature dimension processed by each head, thus limiting the model’s ability to capture comprehensive feature relationships.

The effect of the number of encoder layers shows a similar pattern, as shown in Figure 10B. Model results improve as *N* increases from 1 to 2, with accuracy peaking at 99.48 ± 0.07% and the F1-score reaching 97.60 ± 0.33%. However, adding more encoder layers beyond 2 does not yield significant improvements and shows a slight decline in both metrics. This result indicates that deeper architectures, while potentially capturing more complex patterns, do not necessarily translate to stronger predictive capability for this specific task and may increase computational costs. Based on these observations, we select *H* = 2 and *N* = 2 to balance model outcomes and computational efficiency.

### 4.8. Ablation Study

**Figure 11:**
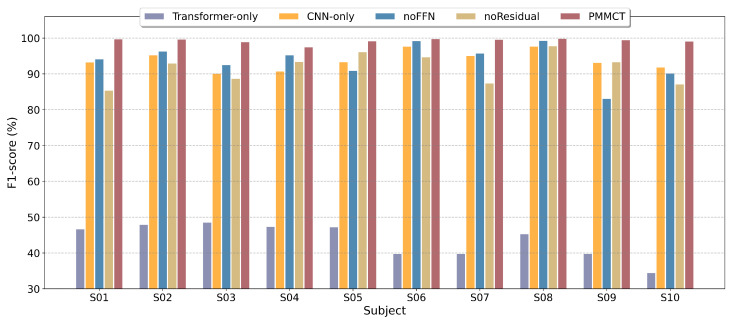
Performance comparison of different ablated models.

We performed ablation experiments under the optimal hyperparameter configuration to assess the contribution of each component. The evaluated variants include a parallel Transformer-only model, a parallel CNN-only model, and models with residual connections or FFN removed. As shown in Figure 11, results show that the full model consistently outperforms all variants across subjects. Importantly, the Transformer-only model exhibits very low performance, indicating that without convolutional feature extraction, the model relies solely on self-attention, which is insufficient to reliably capture local temporal patterns in the raw signals. The CNN-only model shows moderate performance degradation, highlighting the importance of global dependency modeling. Removing residual connections or FFN further reduces trainability and representational capability.

### 4.9. Interpretability Analysis

To address the black-box concern and elucidate the decision basis of the PMMCT model, we visualized the self-attention weights of the Transformer encoder (*N* = 2, *h* = 2) over HEOG and HSUM channels and further computed class-averaged attention maps.

**Figure 12:**
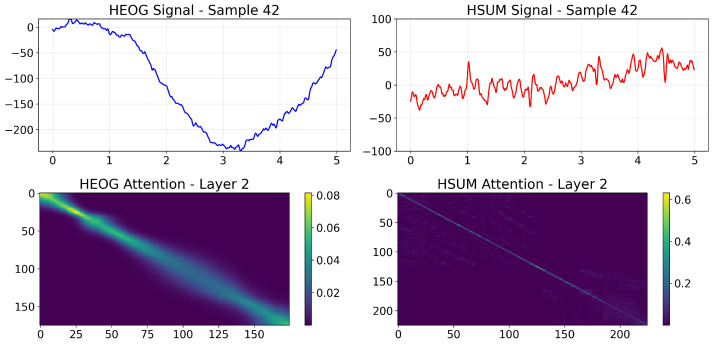
Self-attention visualization for a representative SEM sample. Top: input HEOG and HSUM signals. Bottom: Layer-2 attention maps for each modality.

**Figure 13:**
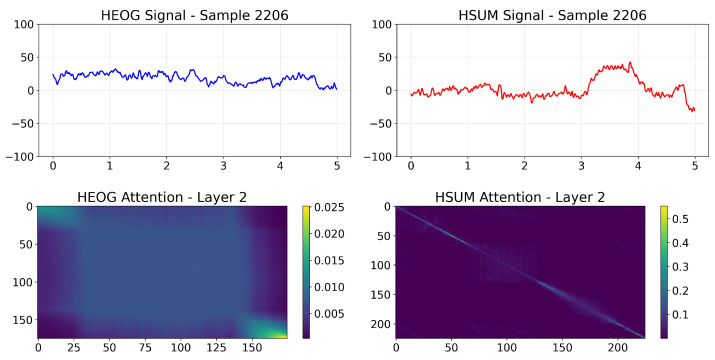
Self-attention visualization for a representative Non-SEM sample. Top: input HEOG and HSUM signals. Bottom: Layer-2 attention maps for each modality.

**Figure 14:**
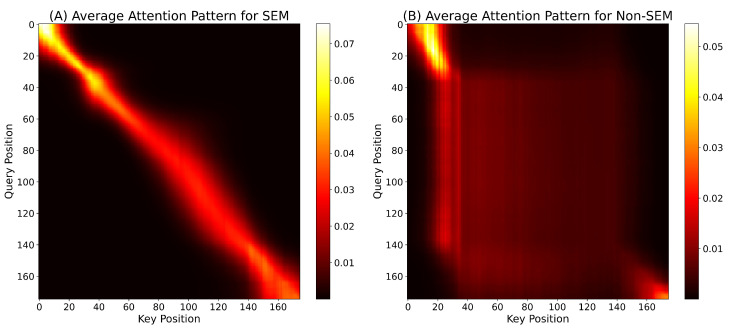
Class-wise average attention maps over the test set. (**A**) SEM class. (**B**) Non-SEM class.

In a representative SEM sample (Figure 12), the layer-2 attention map of HEOG signals shows a strong, continuous diagonal ridge, reflecting long-range integration of sustained, coherent ocular displacements typical of SEM. In contrast, the corresponding map for HSUM signals displays only a weak diagonal pattern, indicating a minor but consistent contribution. In a representative Non-SEM sample (Figure 13), the layer-2 attention map of HEOG signals appears diffuse and lacks a salient diagonal band, while the corresponding map for HSUM signals remains weak without forming any stable pattern. Figure 14 shows the class-wise averaging across all SEM and Non-SEM samples, confirming distinct patterns: the SEM class exhibits a broad, continuous diagonal band, indicating stable global temporal integration, while the Non-SEM class displays an early vertical stripe with otherwise weak and dispersed attention. Overall, PMMCT identifies SEM primarily by aligning sustained, directionally consistent HEOG trajectories over time, with HSUM providing weak but supportive cues, whereas the lack of such long-range structure favors Non-SEM classification.

## 5. Discussion

We adopt an end-to-end deep learning strategy that automatically learns features from raw signals. The proposed PMMCT model combines a CNN module for extracting local eye movement patterns with a Transformer module for modeling global temporal information. Our model attains excellent results with average accuracy and F1-score of 99.89% and 99.35%, while maintaining very low FP and FN, with average values of 5.2 and 0.8, showing high practical value. Notably, the PMMCT model’s effectiveness with the HEOG+HSUM combination demonstrates that reliable SEM detection can be attained using only two single-channel HEOG electrodes, significantly enhancing practicality in driving scenarios. While the model performed well on our 10-subject dataset, we recognize that a larger and more diverse sample is needed to ensure generalizability. The current subject-specific evaluation, where a separate model is trained for each participant, has been used in previous physiological signal studies [20,48] to address inter-subject variability and improve within-subject performance. However, this strategy limits practical deployment, as each new user requires calibration and retraining. Future work will explicitly assess cross-subject generalization to determine whether the model can maintain high performance without per-user retraining and will expand the dataset with participants from varied demographics and driving backgrounds.

In addition, this study has a limitation related to its reliance on manual data annotation, which is time-consuming, may introduce subjective bias, and constrains scalable dataset development. In future work, we will explore semi-supervised or self-supervised learning for automatic annotation to reduce labeling burden and to enable evaluation on larger-scale datasets.

Although the simulator provided a controlled environment for initial validation, real-world complexities such as varying road types, vibrations, environmental noise, and unpredictable driver behavior could adversely affect signal quality and model performance. In response, future work will validate the model under real-road conditions to assess its robustness. Additionally, the two-HEOG-electrode design facilitates wearable integration, which can help reduce motion artifacts and improve practical usability.

## 6. Conclusions

In this study, we propose a Parallel Multimodal CNN-Transformer (PMMCT) model to detect SEMs for recognizing drivers’ SOP. The model employs two parallel feature extraction modules to process bimodal signals, surpassing the unimodal HEOG method, CNN, CNN-LSTM, CNN-LSTM-Attention, and the method relying on manually engineered features. The implementation of the PMMCT model with the HEOG+HSUM combination requires only two single-channel HEOG electrodes, significantly reducing the complexity of wearable devices while maintaining strong predictive capability. We further validated HSUM as an EEG alternative by computing its alpha-band cross-correlation with O2 signals. To enhance interpretability, we visualized Transformer self-attention on HEOG and HSUM, revealing long-range, directionally consistent attentional patterns for SEM and weak, dispersed attention for Non-SEM, with HSUM providing supportive cues. Ablation studies subsequently confirm that the full CNN–Transformer architecture with residual and feed-forward layers consistently outperforms Transformer-only and CNN-only variants, highlighting the importance of both local and global modeling. Overall, this approach demonstrates significant practical value for SEM-based SOP monitoring and facilitates the development of feasible sleepiness monitoring systems. Moreover, we designed the simulated driving experiment and released a physiological signal database, providing a valuable resource for future research on driver sleepiness detection and related applications.

## Figures and Tables

**Table 1:** Test set sample distribution and performance metrics of PMMCT model with optimal hyperparameters.

Subject	Positive Samples	Negative Samples	FP	FN	Precision (%)	Recall (%)	Accuracy (%)	F1-Score (%)
S01	397	2891	1	0	99.75	100	99.97	99.87
S02	454	5627	3	0	99.34	100	99.95	99.67
S03	283	5252	5	0	99.26	100	99.91	99.12
S04	728	6747	34	1	95.53	99.86	99.53	97.65
S05	396	3542	4	1	99.00	99.75	99.87	99.37
S06	193	2756	1	2	99.48	98.96	99.90	99.22
S07	208	3901	1	0	99.52	100	99.98	99.76
S08	637	3158	1	0	99.84	100	99.97	99.92
S09	199	3739	0	2	100	98.99	99.95	99.49
S10	373	4554	2	2	99.46	99.46	99.92	99.46
Average	-	-	5.20 ± 9.71	0.80 ± 0.87	99.12 ± 1.23	99.70 ± 0.40	99.89 ± 0.13	99.35 ± 0.62

**Table 2:** Model performance comparison using grid search: Average scores (mean ± standard deviation) across all parameter combinations in subject-specific evaluation for CNN, CNN-LSTM, CNN-LSTM-Attention, and PMMCT.

	CNN		CNN-LSTM		CNN-LSTM-Attention		PMMCT	
	Accuracy (%)	F1-Score (%)	Accuracy (%)	F1-Score (%)	Accuracy (%)	F1-Score (%)	Accuracy (%)	F1-Score (%)
S01	95.47 ± 2.03	85.36 ± 5.11	87.10 ± 3.83	66.09 ± 7.06	96.87 ± 1.72	89.32 ± 4.92	**98.69** ± 1.01	**95.31** ± 2.63
S02	97.84 ± 2.71	91.42 ± 7.09	96.25 ± 2.14	82.68 ± 6.75	98.72 ± 1.51	94.11 ± 5.18	**99.69** ± 0.45	**98.21** ± 1.78
S03	97.69 ± 1.91	85.01 ± 6.89	94.05 ± 2.57	66.73 ± 8.84	98.75 ± 1.05	90.83 ± 5.58	**99.56** ± 0.33	**96.52** ± 2.23
S04	96.67 ± 1.44	86.41 ± 4.20	96.23 ± 1.37	84.48 ± 4.22	97.85 ± 0.93	90.58 ± 3.28	**98.58** ± 0.59	**93.45** ± 2.38
S05	97.95 ± 1.60	91.90 ± 4.68	95.17 ± 3.02	83.52 ± 7.05	98.39 ± 1.27	93.37 ± 3.79	**99.39** ± 0.49	**97.26** ± 1.86
S06	98.31 ± 1.40	90.67 ± 6.70	96.41 ± 1.99	81.64 ± 6.46	98.76 ± 1.24	92.91 ± 5.49	**99.26** ± 1.19	**96.21** ± 3.65
S07	99.03 ± 1.71	94.67 ± 5.56	97.08 ± 2.89	84.05 ± 7.20	99.11 ± 1.83	95.24 ± 5.09	**99.32** ± 0.90	**95.25** ± 4.02
S08	98.79 ± 0.64	96.48 ± 2.31	97.25 ± 1.50	92.81 ± 3.23	99.10 ± 0.82	97.54 ± 2.04	**99.76** ± 0.28	**99.31** ± 0.75
S09	98.01 ± 1.64	87.20 ± 6.94	96.25 ± 1.78	76.50 ± 7.30	98.76 ± 1.23	91.55 ± 6.09	**99.46** ± 0.73	**96.14** ± 3.65
S10	98.51 ± 1.85	89.43 ± 4.67	96.15 ± 2.13	85.57 ± 5.45	98.93 ± 1.77	92.55 ± 3.69	**99.60** ± 0.59	**97.50** ± 2.01

Note: Bold values indicate the best performance among all models for each subject.

**Table 3:** Optimal hyperparameters of CNN, CNN-LSTM, CNN-LSTM-Attention, and PMMCT models using grid search.

	CNN	CNN-LSTM	CNN-LSTM-Attention	PMMCT
S01	[250, 150, 50, 250]	[250, 150, 250, 250, 150]	[50, 250, 50, 150, 50]	[150, 150, 50, 250]
S02	[250, 150, 50, 150]	[50, 150, 50, 250, 50]	[150, 150, 150, 50, 50]	[250, 50, 50, 250]
S03	[150, 250, 50, 250]	[250, 50, 50, 50, 100]	[150, 150, 50, 50, 50]	[50, 250, 150, 250]
S04	[250, 50, 50, 50]	[250, 150, 50, 250, 150]	[250, 50, 50, 50, 150]	[250, 150, 50, 250]
S05	[150, 50, 50, 250]	[50, 250, 50, 250, 50]	[50, 50, 50, 250, 50]	[50, 50, 50, 150]
S06	[50, 250, 50,250]	[150, 150, 50, 50, 50]	[50, 50, 50, 50, 100]	[50, 250, 50, 50]
S07	[50, 250, 50, 50]	[50, 50, 150, 250, 100]	[150, 250, 50, 150, 50]	[250, 150, 50, 150]
S08	[150, 150, 50, 50]	[50, 250, 50, 50, 50]	[50, 50, 50, 50, 100]	[250, 50, 50, 50]
S09	[150, 50, 50, 50]	[150, 50, 50, 50, 50]	[50, 250, 50, 250, 50]	[50, 250, 150, 150]
S10	[50, 150, 50, 250]	[150, 50, 150, 250, 150]	[250, 50, 50, 150, 50]	[150, 150, 50, 250]

**Table 4:** Test set performance comparison among CNN, CNN-LSTM, CNN-LSTM-Attention, and PMMCT models with optimal hyperparameters.

	CNN		CNN-LSTM		CNN-LSTM-Attention		PMMCT	
	Accuracy (%)	F1-Score (%)	Accuracy (%)	F1-Score (%)	Accuracy (%)	F1-Score (%)	Accuracy (%)	F1-Score (%)
S01	93.73	79.32	98.21	93.03	99.45	97.76	**99.97**	**99.87**
S02	99.77	98.48	98.64	91.62	99.82	98.80	**99.95**	**99.67**
S03	99.78	97.90	95.95	70.13	99.73	97.39	**99.91**	**99.12**
S04	98.98	94.97	94.57	77.86	97.86	90.10	**99.53**	**97.65**
S05	98.81	94.38	90.15	66.32	99.80	99.00	**99.87**	**99.37**
S06	99.73	97.97	99.80	98.47	99.83	98.72	**99.90**	**99.22**
S07	99.03	90.57	99.22	92.59	99.34	93.91	**99.98**	**99.76**
S08	97.47	92.30	99.47	98.45	97.84	93.93	**99.97**	**99.92**
S09	99.52	95.44	96.72	75.43	99.34	93.50	**99.95**	**99.49**
S10	99.63	97.35	99.63	91.25	99.53	96.94	**99.92**	**99.46**
Average	98.64 ± 1.77	93.87 ± 5.44	97.24 ± 2.89	85.52 ± 11.30	99.25 ± 0.72	96.00 ± 2.83	**99.89** ± **0.13**	**99.35** ± **0.62**

Note: Bold values indicate the best performance for each subject.

**Table 5:** Overall performance comparison among Bimodal-LSTM, CNN, CNN-LSTM, CNN-LSTM-Attention, and PMMCT models.

Model	Accuracy ± Std (%)	F1-Score ± Std (%)	FP ± Std	FN ± Std
Bimodal-LSTM	97.96 ± 2.68	89.78 ± 6.99	69.60 ± 21.81	9.40 ± 6.80
CNN	98.64 ± 1.77	93.87 ± 5.44	43.60 ± 56.26	10.00 ± 18.00
CNN-LSTM	97.24 ± 2.89	85.52 ± 11.30	138.70 ± 134.24	7.80 ± 7.63
CNN-LSTM-Attention	99.25 ± 0.72	96.00 ± 2.83	31.60 ± 47.89	3.90 ± 3.73
PMMCT	**99.89** ± **0.13**	**99.35** ± **0.62**	**5.20** ± **9.71**	**0.80** ± **0.87**

Note: Bold values indicate the best performance among all models.

## Data Availability

The datasets generated and analyzed for this study, as well as the code for the proposed CNN-Transformer-based PMMCT model and the comparison experiments, are available in the GitHub repository: https://github.com/DriverSleepinessJiao/SEM_PMMCT (accessed on 8 September 2025).
